# Scientific response to the 2024–2025 dyke intrusions in the Fentale-Dofen Region, Ethiopia: geophysical monitoring, surface manifestations, and hazard mapping

**DOI:** 10.1007/s00445-025-01852-x

**Published:** 2025-07-14

**Authors:** Elias Lewi, Juliet Biggs, Atalay Ayele, Tim Wright, Carolina Pagli, Derek Keir, Yechale Ali, Genet Assefa, Hua Wang, Alessandro La Rosa, Lin Way, Filagot Mengistu, Susan Loughlin, Raphaël Grandin, Tesfaye Temtime, Yelebe Birhanu, Jeffrey Freymueller, Weiyu Zheng

**Affiliations:** 1 Institute of Geophysics, Space Science and Astronomy, Addis Ababa University, Addis Ababa, Ethiopia; 2 COMET, School of Earth Sciences, University of Bristol, Bristol, UK; 3 COMET, School of Earth and Environment, Leeds University, Leeds, UK; 4 Department of Earth Sciences, University of Pisa, Pisa, Italy; 5 School of Ocean and Earth Science, University of Southampton, Southampton, UK; 6 Department of Earth Sciences, University of Florence, Florence, Italy; 7 Ethiopian Geological Institute, Addis Ababa, Ethiopia; 8 College of Natural Resources and Environment, South China Agricultural University, Guangzhou, China; 9 British Geological Survey, Edinburgh, UK; 10 Institut de Physique du Globe de Paris, Paris, France; 11 School of Computing and Engineering, University of West London, London, UK; 12 EarthScope Consortium, Washington, DC USA; 13 Department of Earth and Environmental Sciences, Michigan State University, East Lansing, MI USA

**Keywords:** Northern main ethiopian rift, Dyke intrusion, Seismotectonic activity, Ground deformation, Fentale-dofen, Monitoring

## Abstract

In continental rifts, tectonic deformation, magmatic processes, and earthquakes interact dynamically reflecting the crust’s complex response to extensional stress and evolving subsurface and surface conditions. Recent seismotectonic activity in the Fentale-Dofen region of the Main Ethiopian Rift was driven by the intrusion of several dykes reaching up to ~ 50 km in length observed using satellite radar interferometry. Over 300 earthquakes with magnitude 4 or greater were reported by international seismic networks and the GNSS site at Addis Ababa moved ~ 20 mm to the west. These and other observations on the ground were used to create a highly simplified hazard map and 75,000 people were evacuated. Although no magmatic eruption occurred, the earthquakes triggered landslides and caused infrastructure damage, especially to buildings and roads. Here we provide a preliminary analysis of the patterns of earthquakes, ground deformation, and surface manifestations from 2024 to 2025, with a focus on the underlying mechanisms contributing to seismic sequences in the area and key unresolved scientific questions. We discuss how scientific evidence was used to inform decision-makers and examine the short- and long-term implications for critical infrastructure and nearby communities. Finally, we emphasize the importance of real-time monitoring, proactive risk management, and the need for continuous observation and improved early warning systems to reduce future seismic and volcanic risks.

## Introduction

Dyke intrusions are common in extensional tectonic settings, including rift systems and the flanks of volcanic islands, often culminating in fissure eruptions (Neal et al. 2019; Parks et al. 2025; Sigmundsson et al. 2015; Wright et al. 2006). In well-monitored regions, dense networks of seismometers and GNSS instruments track intrusions in real-time and there has been some success in forecasting eruptions for example (Parks et al. 2025). In contrast, the 2021 eruption of Mount Nyiragongo in the Democratic Republic of the Congo serves as a timely reminder of the consequences of dyke intrusions and eruptions occurring in densely populated areas with little warning (Smittarello et al. 2022).

**Fig. 1:**
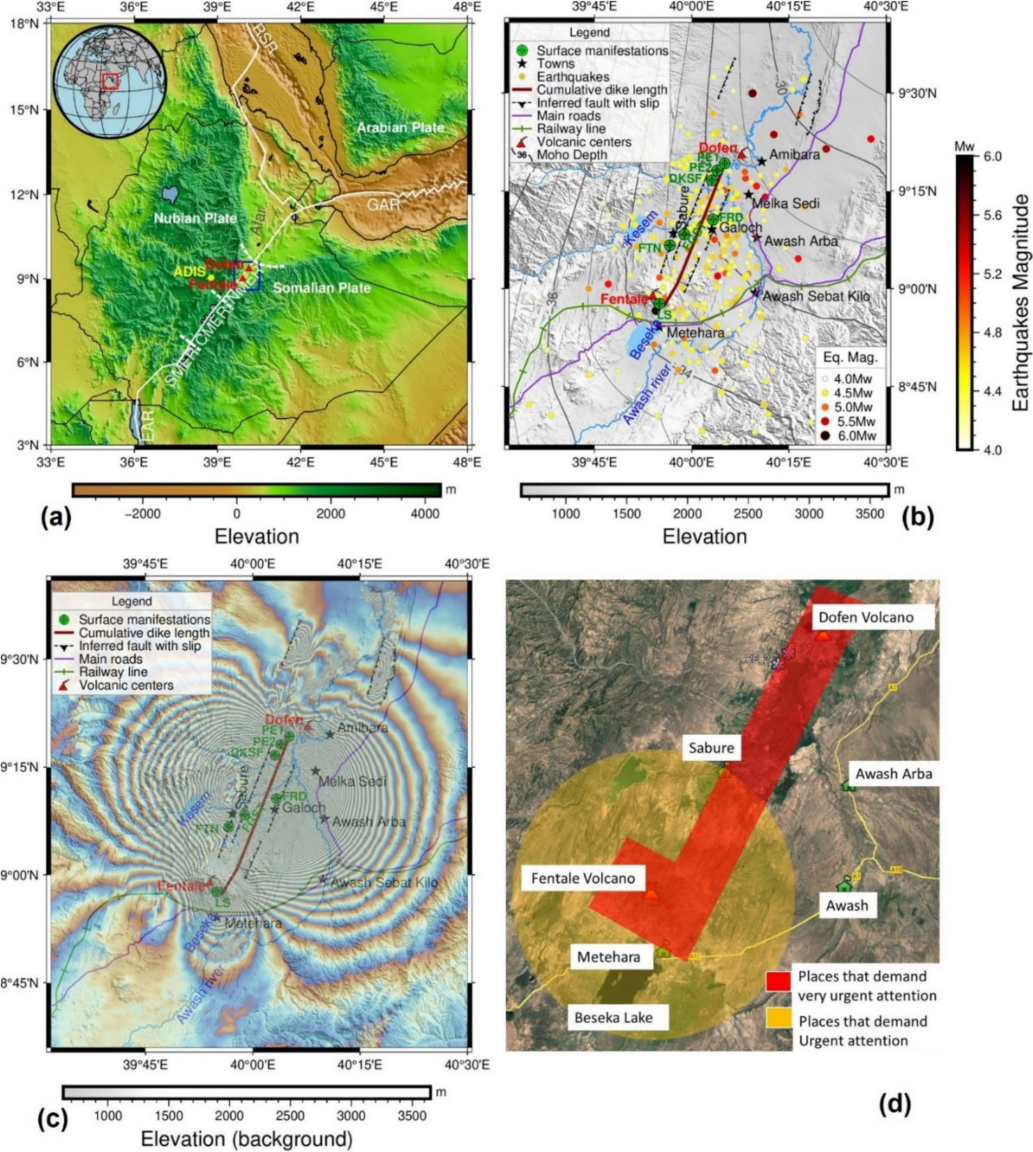
Maps of the Fentale–Dofen volcanic region, Northern Main Ethiopian Rift. **a** Regional map showing the location of the Fentale and Dofen volcanic centres within the broader tectonic setting of the Northern Main Ethiopian Rift. The map includes major tectonic features and indicates the relative position of Addis Ababa. The blue square outlines the area detailed in **b**–**d**. **b** Detailed map of the Fentale–Dofen area displaying topography, the locations of volcanic centres, major towns, surface manifestations, the extent of cumulative dyke intrusions, earthquake epicentres, interpreted faults, and key infrastructure such as roads and railways. The contour lines show the depth to the Moho (Lewi 2024). **c** Interferogram derived from Sentinel-1 InSAR data, showing surface displacement between 17 December 2024 and 22 January 2025. Each coloured fringe represents a displacement of 28 mm in the satellite line-of-sight (LOS). **d** A simplified volcanic hazard map developed and utilized during the crisis period, indicating zones of varying volcanic hazard based on observed and inferred geological and geophysical data

Between September 2024 and March 2025, a sequence of dyke intrusions occurred in the densely populated Northern Main Ethiopian Rift. The intrusion occurred between Fentale and Dofen volcanoes, located about 145 km east of Addis Ababa. Due to the prevailing conditions in the region, access was restricted, and ground-based monitoring was not feasible. Various organizations, including the Ethiopian Geological Institute, visited parts of the area and inspected the damage to buildings and roads from September 2024 onwards. Real-time earthquake locations were determined by the Institute for Geoscience, Space Science and Astronomy (IGSSA) at Addis Ababa University (AAU) using three regional stations: AAE in Addis Ababa, FURI located 18 km SSW of Addis Ababa, and ATD in Djibouti. GNSS data were recorded and processed by IGSSA at the International GNSS Service (IGS) site ADIS in Addis Ababa to assess ground motion. These stations are located 142 km WSW (ADIS and AAE), 154 km WSW (FURI), and 403 km NE (ATD) from Galoch, a village in the central part of the dyke axis (Fig. 1b). Satellite Interferometric Synthetic Aperture Radar (InSAR) data was tasked to increase the frequency of acquisitions (every 12 days from Sentinel-1 and every 1–8 days from COSMO-SkyMed) and processed by several universities internationally.

In this short scientific communication, we describe how scientific evidence was used to inform decision-makers and examine the short- and long-term implications for critical infrastructure and nearby communities. We then discuss the broader geohazard context, emphasizing the importance of real-time monitoring, proactive risk management, and the need for continuous observation and improved early warning systems to reduce future seismic and volcanic risks.

## Background

The Fentale-Dofen region is seismically and volcanically the most active part of the Northern Main Ethiopian Rift (Keir et al. 2006; Wadge et al. 2016), located close to the Triple Junction between the Red Sea, Gulf of Aden, and East African Rifts (Fig. 1). The region is characterized by active tectonic processes, including continental rifting, volcanism, faulting, and associated earthquakes. The rate of extension in the Northern Main Ethiopian Rift is ~ 5–6 mm/year (Kogan et al. 2012; Saria et al. 2014). There is a steep gradient in Moho depth along the Northern Main Ethiopian Rift (Fig. 1b), associated with the transition between the > 40-km-thick crust of the Ethiopian Plateaux and < 20-km-thick crust in the Afar Depression (Lewi 2024).

The geology of the Fentale area is dominated by silicic volcanic rocks, including rhyolite, obsidian, and pantellerite. Trachytic flows are present in the older layers and basaltic rocks in the Fentale area limited to the southwestern side (Fontijn et al. 2018; Gibson 1974). Similarly, the Dofen Volcanic Center (DVC), which is located 47 km northeast of Fentale, primarily features felsic volcanic rocks, including peralkaline rhyolites and pantellerites that form the main volcanic edifice (Chernet 2005). The early phases of volcanic activity in the region were marked by the extrusion of trachybasalts, and recent activity has involved the eruption of basaltic lava flows and scoria cones, reflecting ongoing mafic volcanism.

Historical reports date the basaltic lava flows on the southern flank of Fentale to around 1810 (Williams et al. 2004) and seismic swarms were recorded in 1981, 1989, 2003, 2004, and 2015 (Ayele et al. 2024; Temtime et al. 2020). Geodetic imaging of the 2015 swarm showed that it was driven by the intrusion of a 6-km-long dyke located to the NE of Fentale’s caldera, with seismicity extending to a depth of 40 km (Ayele et al. 2024; Temtime et al. 2020).

Fentale is located ~ 127 km air distance east of Addis Ababa, about 7.5 km north of the main asphalt road and railway route that connects Ethiopia and Djibouti (Fig. 1a). The towns located within a 20-km range of the active Fentale-Dofen Region (Fig. 1b) have a combined population of ~ 100,000 according to the Ethiopian Statistical Service (2023) including Metehara (20,582), Awash Sebat Kilo (38,447), Awash Arba (13,027), Sabure (5,092), Amibara (10,000), and Melka Sedi (14,215). Galoch is a small village located at the mid-segment of the 50-km-long dyke, established to accommodate the staff of the Kesem Sugar Factory.

## Evolution of the Fentale–Dofen crisis

The Fentale–Dofen crisis unfolded between September 2024 and April 2025 is marked by alternating periods of seismic activity, dyke intrusion, faulting, surface deformation, and intervals of relative quiescence. To facilitate interpretation and better understand the events, it is divided into four phases based on changes in seismicity, geodetic signals, and surface deformation, observed in the area.

### Phases 1 and 2: september–december 2024

**Fig. 2:**
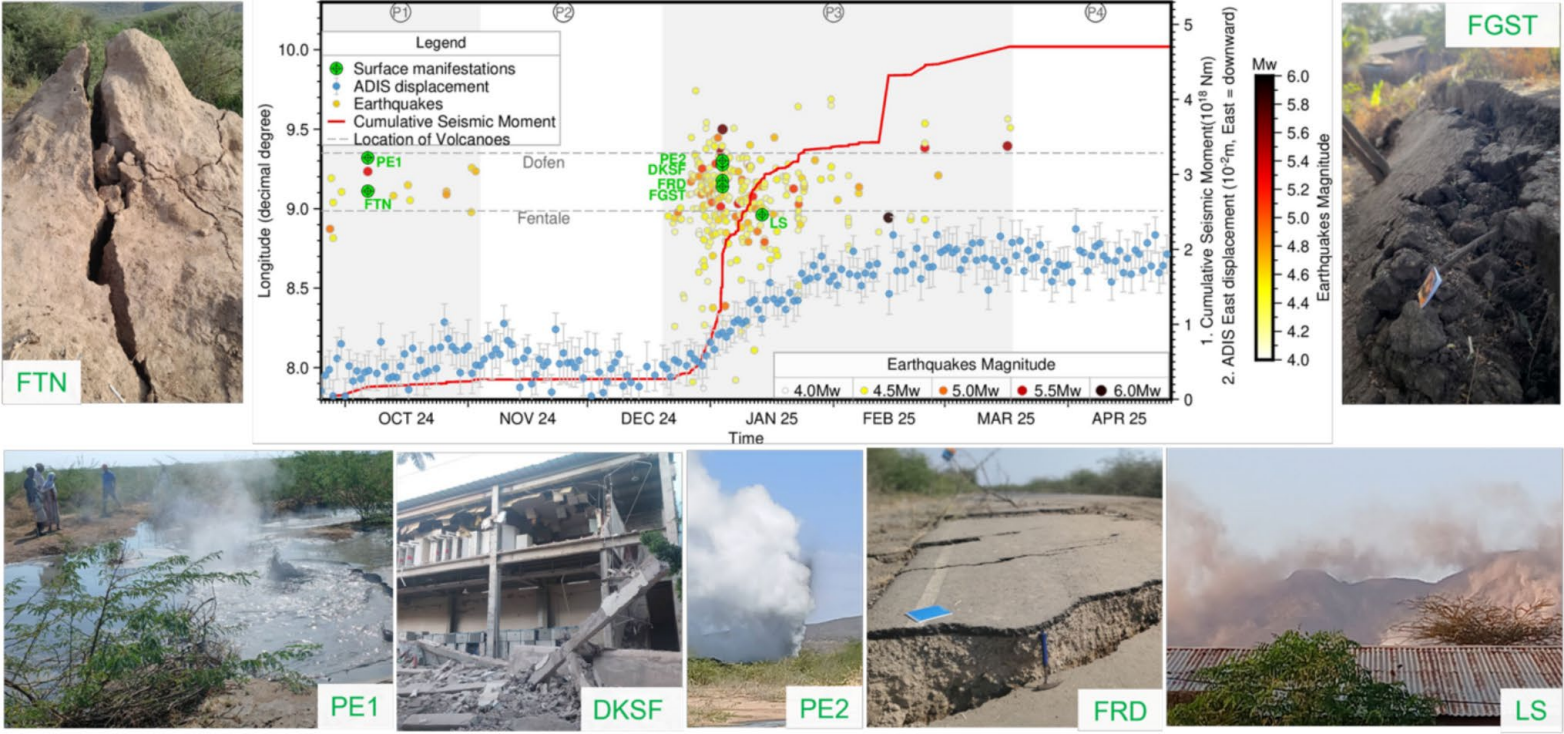
Timeline of the activities during the 2024–2025 Fentale-Dofen tectonic–magmatic event, integrating seismic, geodetic, and field observations to provide a comprehensive overview of the dynamic surface response to the evolving dyking process and associated seismicity. The central panel shows the latitude of seismic events (primary y-axis) plotted against time (x-axis), illustrating the migration and clustering of earthquakes. Superimposed on the plot are two additional y-axes: the cumulative seismic moment release (right-side y-axis 1) tracking the progressive buildup of seismic energy, and the displacement of the GNSS station ADIS, which is an IGS station (right-side y-axis 2). To isolate the anomaly, ADIS’s expected position was estimated by extrapolating a linear trend fitted with data up to early September 2024, and the predicted trend was deducted from the processed data. Surface manifestations associated with the seismic sequence are represented by symbols at corresponding latitudes and times, including damaged buildings (DKSF), surface ruptures (FTN, FRD, FGST), phreatic eruptions (PE1, PE2), and landslides (LS)

A series of earthquakes began in the Fentale-Dofen region in late September 2024, including tremors over magnitude 4 on September 27, 2024 (Phase P1, Fig. 2). A stronger quake, with a magnitude of 5.3, occurred on October 6, 2024, at 17:10:04 GMT and was felt as far away as Addis Ababa, which is home to about 4 million people (Ethiopian Statistical Service 2023). The earthquake was especially felt strongly in the eastern part of Addis Ababa, about 110 km from Galoch. This prompted many residents of Addis Ababa and other towns to temporarily leave their homes, uncertain of potential aftershocks, as such a strong and widely felt earthquake had not been experienced by the population in recent memory. People remained outdoors until the epicentre was located by IGSSA and communicated to the public. Earthquakes including those with magnitudes greater than 4 were felt continuously through November 2, 2024.

A Sentinel-1 satellite image showed up to 17 cm of deformation in the satellite line-of-sight in a butterfly-shaped pattern typical of dyke intrusions (Keir et al. 2025; Way et al. 2025) confirming that the earthquakes were generated by subsurface magmatic activity near Fentale. The GNSS station ADIS, which is situated around 142 km west of Galoch, started to show a little anomalous displacement on September 17, 2024, departing from its usual eastward motion in the ITRF14 reference frame. The displacement exactly matched the start of Phase One (P1) of the event, even though it was still within the station’s error limits, which may have typically been interpreted as atmospheric or other local effects (Fig. 2).

The first noticeable surface manifestations were reported following the magnitude 5.3 earthquake on October 6, 2024 (Figs. 1 and 2). Ground fractures started to manifest themselves in the southern part of the area, northwest of Fentale, and a phreatic eruption (PE1) occurred to the southwest of Dofen. One of the clearer illustrations of fracture formation can be seen at a site where a termite nest is transected by surface fractures (FTN). The cause of the earthquake, the history of volcanism and seismic activity of the area, and the potential risks were communicated directly to government agencies and shared with the public through the media.

This initial period of unrest (P1) was then followed by a quiescent phase (P2), as shown in Fig. 2, during which no earthquakes were recorded, no surface deformation was detected by InSAR imagery, and no reports of surface manifestations were received. The tremors remained calm during phase P2 (Fig. 2) until December 19, 2024, and the GNSS station ADIS resumed its northeast migration until December 22, 2024.

### Phases 3 and 4: december 2024–april 2025

Earthquake activity at Fentale-Dofen resumed on December 20, 2024, with a sharp increase in the number and magnitude of events detected, many of which were felt as far away as Addis Ababa. A Sentinel-1 InSAR image acquired on December 29 indicated a dyke intrusion extending from near Metehara to the south of Dofen showing that the intrusion had reached a length of ~ 50 km (Fig. 1b). The dyke intrusion was accompanied by slip on normal fault systems (Fig. 1b and c), a network of surface offsets, and subsidence at Fentale (Way et al. 2025). High-resolution COSMO-SkyMed data showed details of the rapid dyke propagation, reaching Dofen, located 47 km to the north of Fentale, by the 29th of December (Way et al. 2025).

In the following days, hundreds of tremors were recorded daily at IGSSA. On January 3 and 4, 2025, over 35 earthquakes exceeding magnitude 4 were recorded within 48 h along the cumulative dyke intrusion length (Fig. 2). The largest of these was a magnitude 5.7 event north of Dofen, accompanied by three other earthquakes with magnitudes equal to or greater than 5.0. As shown in Figs. 1 and 2, these seismic events, particularly the magnitude 5.7 earthquake, were spatially and temporally associated with several significant surface manifestations. These among others include the structural damage to part of the Kesem Sugar Factory building, about 0.35-m vertical displacement (downthrow) of a fracture crossing an asphalt road (FRD), the appearance of a second phreatic eruption (PE2) near Dofen, and around 1 m of tilting and downthrow of another fracture (FGST) north of Fentale. The fault designated as FRD in Figs. 1 and 2 dips steeply at 83° toward a dip direction of 290°, corresponding to a strike of approximately N20°E. Similarly, the FGST fault dips steeply at 85° toward a dip direction of 110°, also exhibiting a strike near N20°E. These fault orientations align with the regional extensional framework and are consistent with InSAR and seismological data confirming the formation of normal faulting. Between January 11, 2024, and February 20, 2025, several major earthquakes occurred, primarily in the southern part of the region near Fentale, including two events with magnitudes exceeding 5.0. These seismic events coincide both temporally and spatially with a landslide in the southeastern sector of the Fentale volcanic centre (Figs. 1 and 2).

By December 29, 2024, 176 homes and small buildings had been damaged, mostly by cracked or fallen walls, but the structures themselves were still intact. Moreover, the walls of seven schools, nine healthcare facilities, three agricultural centres, and three religious institutions have been reported to exhibit cracks varying from slight to severe. However, on January 4, 2025, it was reported that the Kesem Sugar Factory suffered partial structural damage as a result of the January 3 and 4 earthquakes (DKSF, Fig. 2). The recurring nature of these seismic events prompted discussions among local officials and the public about potential risks and necessary precautions. The frequency and intensity of the tremors, along with their reach to urban centres, underscored the urgency of addressing the situation proactively.

The GNSS station in Addis Ababa, ADIS, showed considerable westward movement starting around December 27, 2024, and lasting until January 26, 2025. By February 20, the GNSS station had shifted approximately 20 mm westward from its expected position based on its typical north-eastward motion. The cumulative seismic moment and the GNSS displacement pattern are in good agreement, except for February 14, 2025, when a magnitude 5.9 earthquake created a significant increase in seismic moment without significant surface displacement measured by GNSS at Addis Ababa. Global seismic networks show that this earthquake had an unusual focal mechanism and previous studies have attributed similar mechanisms to motion along curved ring faults associated with caldera collapses (Gudmundsson et al. 2016; Shreve et al. 2022; Shuler et al. 2013). Multiple satellite observations show localized collapse within the Fentale caldera during the same period (Way et al. 2025). On this occasion, the GNSS station displayed disturbance, but the trend remained unchanged. Given that there was no observed surface deformation and no ground shaking experienced by anyone in the immediate area or Addis Ababa, it is likely that this earthquake occurred at a higher depth and did not result in noticeable surface deformation.

Satellite InSAR data show that between early January and mid-March, the dyke continued opening, without propagating (Way et al. 2025). The rate of dyke opening was variable in space and time but showed an overall decrease. The correlation between dyke opening, subsidence to the SW of Fentale, and seismic moment release suggests that the dyke may be fed by a source near this subsiding area SW of Fentale (Way et al. 2025), but the involvement of a deeper source cannot be discounted.

Although the most dramatic activity has occurred along the rift axis between Fentale and Dofen, there have also been signs of reawakening at Fentale itself. Starting on 13–14 January, in addition to localized deformation, thermal and gas anomalies were detected within the caldera at Fentale, consistent with enhanced fumarolic activity and release of deep-seated gases (Way et al. 2025). Similar observations at Agung volcano, Indonesia, preceded a phreatomagmatic and then magmatic eruption which began 2 months after a dyke intrusion between Agung and its neighbour, Batur (Bemelmans et al. 2023).

The rate of seismicity and surface deformation in the Fentale-Dofen area has been low since March 17, 2025 (Phase P4), but it is unclear how long this period of quiescence will last.

## Hazard map and evacuation

A scientific advisory committee comprising colleagues from IGSSA and the School of Earth Science (SES) at AAU, the Ethiopian Geological Institute (EGI), and other universities and institutions met on 2 January to review all available information. While the potential risks were deeply concerning, it was also necessary to avoid causing unnecessary panic or raising false alarms. The preparation of a formal volcanic hazard map or event tree would require considerable time, input, and fieldwork, which was not feasible. Instead, the committee provided a highly simplified zoned map summarising the most likely scenarios.

If an eruption were to occur, a basaltic fissure eruption between Fentale and Dofen was considered the most likely scenario. The area marked in red in Fig. 1 (“Places that demand very urgent attention”) spans a width of 8 km, covering 4 km on either side of the interpreted dyke intrusion zone. While the potential for the flow of mafic magma over larger distances from fissure eruptions was recognized by the committee members, the 4 km buffer was chosen considering the significant economic resources required if evacuation is to be made. The 4-km buffer zone was intended to allow time to initiate life-saving efforts.

Less emphasis was placed on an eruption at Fentale because satellite imagery showed that the area was deflating. Fentale was included due to concerns about potential landslides and the collapse of the volcanic centre due to deflation. The alignment of the dyke intrusion with Metehara meant that any unforeseen shift in the propagation direction from NNE to SSW could affect the area. Therefore, both Metehara and Fentale were still designated as “Places that demand very urgent attention” (Fig. 1d). A 20-km radius around the summit of the Fentale Caldera, marked in yellow (Fig. 1d), was designated as “Places that Demand Urgent Attention”. This was used as a no-fly zone to reduce the risk to aviation of an unexpected volcanic eruption at the caldera summit, which could directly impact flights over the mountain. This measure also provides time to issue additional directives if such an event occurs.

This simplified hazard map, prepared under challenging circumstances, was then submitted to government offices for use in any actions the government may take. The United Nations Office for the Coordination of Humanitarian Affairs reported that the Government, supported by UN and NGO partners, evacuated approximately 75,000 people (approximately 20,000 in Oromia and 55,000 in Afar) to safer locations (OCHA 2025). There have been no documented human casualties associated with the crisis thus far.

## Discussion

There are several unresolved questions regarding the Fentale-Dofen area, particularly concerning the ongoing volcanic activity and dyke intrusions. Key questions include whether the dyking episode will continue and if a volcanic eruption is likely. It remains unclear whether the dyke intrusions are directly fed by the magma source in the southwest or if a deeper magma chamber connects both areas. The southwestern part of Fentale has a history of mafic eruptions (Fontijn et al. 2018; Gibson 1974) and is currently undergoing deflation, while the northeastern part is inflating, with the dyke propagation rate suggesting mafic involvement (Keir et al. 2025). This has led to the hypothesis that the magma fuelling the northeastern dyke intrusion may come from the southwestern region. However, a key issue is whether both regions are drawing magma from the same deeper reservoirs and whether the deflation in the southwest is due to magma being redirected to the northeast via the deeper magma chamber. This remains an important area for further study.

While seismicity and deformation have been low since March 2025, many dyke intrusions typically occur in sequences lasting many years to decades, with pauses that range from a few months to several years (Hamling et al. 2010; Montgomery-Brown and Miklius 2021). The time lapse between diking episodes can vary widely depending on the geological setting and the type of magma involved. The classic models of Buck et al. (2006) predict that (1) the first dyke in each sequence should be the longest followed by successively shorter dykes and (2) when tectonic stresses have been largely relieved, then extrusion of magma may start (Buck et al. 2006). On initial inspection, the pattern at Fentale does not appear to fit this pattern; the dyke intrusion in 2015 was ~ 6 km long (Temtime et al. 2020) and the Sept–Oct 2024 intrusion was ~ 14 km long (Keir et al. 2025; Way et al. 2025) and the most recent (Dec 2024-March 2025) was ~ 50 km long. However, the 2015 intrusion was not well-aligned with the active fault system or extensional direction (Temtime et al. 2020) suggesting that the topographic stress field from Fentale’s edifice is locally dominant (Wadge et al. 2016). Similarly, the Dec 2024–March 2025 initially propagated radially to Fentale before aligning with the regional stress field as it propagated away from the volcano (Way et al. 2025), following the segmented propagation pattern observed during the 2014–2015 dyke intrusion at Bardarbunga, Iceland (Sigmundsson et al. 2015). Therefore, if we assume that the 2025 dyke is the first along the Fentale-Dofen rift axis where the dominant regional tectonic stresses dominate, we might expect the predictions of Buck et al. (2006) to hold. Specifically, there will be a sequence of dyke intrusions along the rift axis between Fentale and Dofen, each one with decreasing length and increasing lava extrusion. Furthermore, the ongoing thermal, deformation, and gas anomalies at Fentale, persisting at least through May 2025, could be indicative of the potential for an explosive, felsic eruption, suggesting the possibility should not be ruled out. Neither can we discount the possibility that the dyke will propagate south of Fentale, once the tectonic stresses between Fentale and Dofen are relieved.

Investment in disaster risk reduction is therefore a priority. The United Nations Office for Disaster Risk Reduction states that every US$1 invested in risk reduction and prevention can save up to US$15 in post-disaster recovery. In any geohazard crisis, scientists and officials are required to make decisions under conditions of extreme uncertainty, both due to the stochastic nature of the problem and the unavoidable epistemic uncertainties (Doyle et al. 2014). Improving local monitoring networks and understanding the interconnected nature of the magmatic plumbing system is therefore a priority (Biggs et al. 2021; Temtime et al. 2020). State-of-the-art lava flow hazard maps use a statistical approach to integrate potential vent sites with numerical models to create probabilistic maps of lava flow inundation areas (e.g. Wadge et al. 1994; Zuccarello et al. 2023). In the past few years, the very first probabilistic hazard assessments for volcanoes in Ethiopia have been produced (Clarke et al. 2020; Tierz et al. 2020) and these provide a useful framework for future work in the Fentale-Dofen region. Potential cascading hazards must also be considered, such as possible impacts on downstream agriculture caused by disruption of the Awash River or the impact on supply chains and food security if the asphalt road linking Addis Ababa to Djibouti was impassable. Considering Ethiopia’s vulnerability to seismic and volcanic hazards, there is an opportunity to further strengthen the country’s preparedness and response capabilities. While the Ethiopian Disaster Risk Management Commission (EDRMC) has made significant progress in addressing droughts and floods, there may be potential for enhancing systems specifically tailored to managing earthquakes and volcanic activity. There is an opportunity to further refine protocols and reporting mechanisms for these specific risks, which could improve early warning systems and overall crisis management efforts. Additionally, there are opportunities to further enhance the organization and timeliness of response efforts, emphasizing the importance of improved coordination and resource allocation. Ensuring the safety of critical infrastructure also requires more attention, as construction practices that may not fully incorporate scientific input could lead to considerable challenges for the nation during natural disasters. Strengthening Ethiopia’s systems for monitoring, reporting, and responding to these risks would not only bolster public safety but also safeguard vital infrastructure and foster more effective crisis management overall.

## Conclusions

In conclusion, the seismic activity observed between September 26, 2024, and March 17, 2025, was associated with successive dyke intrusions extending approximately 50 km from the eastern foot of Fentale to the Dofen volcanic centre, with a pause from November 2 to December 20. A significant earthquake swarm on January 3–4, particularly the magnitude 5.7 event, caused damage to infrastructure such as buildings and roads. Surface observations revealed that these earthquakes are associated with vertical displacements of up to 1 m along faults and caused partial structural damage to the Kesem Sugar Factory building. In addition, the landslide in the southeastern part of the Fentale area is associated with the earthquakes that occurred between January 11 and 14. Apart from this, the intrusions, especially those after December 20, caused significant surface displacement, including a westward shift of the GNSS station ADIS, located 144 km away. Ethiopia’s susceptibility to seismic and volcanic hazards underscores the urgent need for enhanced scientific monitoring and preparedness. This situation acts as a wake-up call, highlighting that not only areas within the rift but also towns like Addis Ababa and infrastructure along the rift margins are vulnerable to seismo-tectonic events.

The ongoing volcanic activity in Fentale, characterized by dyke intrusions and the observed patterns of deflation and inflation, raises several critical questions: Will the dyking episode continue, and is a volcanic eruption imminent? The southwestern part of Fentale, known for its history of mafic eruptions and current deflation, contrasts with the northeastern part, which is experiencing inflation and mafic dyke propagation. This points to the possibility of magma transfer from the southwest to the northeast, but it remains unclear whether both regions are drawing magma from a shared deeper reservoir. The deflation observed in the southwest could be due to magma being redirected toward the northeast, yet this interaction requires further investigation. Addressing these scientific uncertainties is vital for strengthening Ethiopia’s ability to manage seismic and volcanic risks effectively.

To effectively manage seismo-volcanic risks in Ethiopia, it is crucial to prioritize the enhancement of scientific monitoring and research to improve understanding of seismotectonic events. Investments should focus on strengthening human, infrastructural, and institutional capacities to support these efforts. Enhancing early warning systems and refining reporting protocols will ensure more timely responses to emerging threats. Additionally, fostering better coordination between scientific institutions, government agencies, and local communities is essential for efficient crisis management. Ensuring the safety of critical infrastructure by strengthening the integration of scientific findings into construction practices will help minimize the impact of volcanic events. Continued investment in research is vital for addressing existing uncertainties and strengthening long-term preparedness for seismic and volcanic hazards. Moreover, providing media professionals with proper training on geohazard reporting is crucial to avoid public panic and ensure accurate, responsible communication during volcanic and seismic emergencies.

## Data Availability

Processed Sentinel-1 InSAR data is available through the COMET Volcanic and Magmatic Deformation Portal: https://comet.nerc.ac.uk/comet-volcano-portal/ Seismological data used in this study were obtained from the GEOFON seismic network data archive, hosted by the Helmholtz Centre for Geosciences (GFZ), Potsdam. These datasets are publicly accessible and can be retrieved from https://geofon.gfz.de/eqinfo/list.php. GNSS data for the station ADIS, which is maintained by the Institute of Geophysics, Space Science and Astronomy (IGSSA) of Addis Ababa University, along with data from other GNSS stations used as a reference, were obtained from the Crustal Dynamics Data Information System (CDDIS). These data sets are publicly accessible and can be retrieved from https://cddis.nasa.gov/. This research has utilized a Google Earth satellite image (https://earth.google.com) as a basemap for visualizing the volcanic hazard map. GNSS data were processed using the GAMIT/GLOBK software package developed by the Massachusetts Institute of Technology (Herring et al 2010). Most figures were generated using the Generic Mapping Tools (GMT), an open-source software suite for geospatial data processing and visualization (Wessel et al. 2019).
